# Medical educators’ perceptions of race in clinical practice

**DOI:** 10.1186/s12909-024-05232-5

**Published:** 2024-03-04

**Authors:** June Futterman, Catherine Bi, Brendan Crow, Sarah Kureshi, Ebiere Okah

**Affiliations:** 1 Children’s National Hospital, Washington, DC USA; 2 Cedars-Sinai, Los Angeles, CA USA; 3 Mountain Area Health Education Center, Asheville, NC USA; 4 Georgetown University School of Medicine, Washington, DC USA; 5 Family Medicine and Community Health, University of Minnesota Medical School, Minneapolis, MN USA

**Keywords:** Race-based medicine, Medical curriculum, Medical educators, Structural racism, Racial inequity

## Abstract

**Background:**

While several medical societies endorse race as a social construct, it is still often used as a biological trait in medical education. How medical educators employ race while teaching is likely impacted by their beliefs as to what race represents and its relevance in clinical care. Understanding these beliefs is necessary to guide medical education curriculum reform.

**Methods:**

This was a qualitative survey study, conducted in June 2020, of Georgetown University Medical Center faculty. As part of the survey, faculty were asked to rate, on a 5-point Likert scale, the extent to which they perceived race as a biological trait and its importance in clinical care. Self-identified clinical or preclinical faculty (*N* = 147) who believed that race had any importance were asked to provide an example illustrating its significance. Free-text responses were coded using content analysis with an inductive approach and contextualized by faculty’s perspectives on the biological significance of race.

**Results:**

There were 130 (88%) responses categorized into two major themes: race is important for (1) screening, diagnosing, and treating diseases and (2) contextualizing patients’ experiences and health behaviors. Compared to faculty who perceived race as biological, those who viewed race as strictly social were more likely to report using race to understand or acknowledge patients’ exposure to racism. However, even among these faculty, explanations that suggested biological differences between racial groups were prevalent.

**Conclusions:**

Medical educators use race primarily to understand diseases and frequently described biological differences between racial groups. Efforts to reframe race as sociopolitical may require education that examines race through a global lens, accounting for the genetic and cultural variability that occurs within racial groups; greater awareness of the association between structural racism and health inequities; movement away from identity-based risk stratification; and incorporation of tools that appraise race-based medical literature.

## Background

Despite several medical societies recognizing race as a sociopolitical construct incapable of adequately capturing human genetic variation [1–3], medical educators often treat it as a biological factor. For example, Tsai et al. [4] found that 96% of preclinical curriculum slides suggested biologic risks associated with race. While medical students begin their training with their own perspectives on race [5], how medical educators teach race still affects these learners by shaping their perceptions of when race is relevant in care, which, in turn, has the potential to impact how these learners practice over the course of their careers [6]. As such, current efforts to reframe race as a social phenomenon requires collaboration with medical educators and should be guided by an understanding of why educators find race useful in clinical decision-making.

Few studies have examined how medical educators conceptualize race and their views regarding the utility of race when making decisions about patient care. A 2020 survey **by** Ibrahim and colleagues found that most pre-clinical educators viewed race as a social construct [7]. In contrast, a 2010 survey of internal medicine physicians found that 81% believed race represented genetic ancestral or biological groups; only 16% described racial groups as sociocultural [8]. Differences between the findings of these two studies can be attributed to several factors including an evolution in how race is perceived in general society along with advocacy against race-based (incorporating race in clinical decision-making processes) clinical practice within medical institutions [1–3]. However, in the aforementioned study by Ibrahim et al., the researchers also found that despite a high proportion of pre-clinical educators describing race as socially determined, in lecture materials used to teach first-year medical students, race was used without context or depicted as a marker of biological risk, similar to the findings of Tsai et al., [4] and revealing a potential discrepancy between the reported beliefs of medical educators and their observed actions when teaching medical students. More needs to be known as to how educators utilize race, particularly within the context of their perceptions of race as a social or biological phenomenon.

As an extension of the work done by Ibrahim and colleagues, we sought to qualitatively explore the relationship between medical educators’ view of race as a biological phenomenon and their beliefs regarding its relevance in clinical care. We aimed to gain insight into why race is taught as a marker of disease risk, taking into account educators’ perceptions of race as biological or social in nature.

## Methods

### Study design

This was a mixed methods study comprised of closed and open-ended survey items. An email invitation containing a link to an online survey was sent to all medical faculty affiliated with Georgetown University Medical Center on June 22, 2020. The survey remained open until July 13, 2020, and recipients were given one email reminder during this time. Participants were required to fully complete each page of the survey before moving to the next portion, but survey completion was not required for submission. No incentives were given for study participation. This study was approved by Georgetown University Institutional Review Board.

Participants were asked to complete a 12-item questionnaire evaluating their perceptions of race in clinical care. Items assessed regarded opinions on biological race, the importance and comfort with discussing race in medical education, and how conducive the environment at Georgetown University School of Medicine was for having open conversations about race in medicine. Most responses were scored on a five-point Likert scale (1 = not at all important, 5 = extremely important).

### Study population

There are 4,032 faculty on the Georgetown University Medical Center and Georgetown University Faculty listservs, of which 281 completed the survey. The study sample (*N* = 147) was limited to preclinical or clinical faculty who responded that race was at least slightly important (Likert scale score 2 to 5) when asked “How important is it to consider race when making clinical decisions?”

### Study question

We evaluated the free-text response to the question “Could you describe or give an example of how race is important to clinical decisions?” Participants were given 20,000 characters to respond. Responses were contextualized by participants’ answers to the question “To what extent is there a biological basis for race?” (scored on a five-point Likert scale: 1 = not at all, 5 = to a great extent), where appropriate.

### Demographic characteristics

Using minimum federal census categories, participants selected a racial identity (African American or Black, American Indian or Alaska Native, Asian, or White), ethnicity (Hispanic/Latino/Spanish, Not-Hispanic/Latino/Spanish), gender identity (female, male), and faculty role (pre-clinical, clinical, and/or research). All demographic questions were given the option of “other” as a response, with free text entry to allow for participants to best self-identify. Of note, faculty who defined their role as strictly research or “other” were not included in the study sample.

### Analysis

We analyzed the survey responses using content analysis with an inductive approach. Coding was conducted in Excel version 16.54 (Microsoft, Washington DC) and transferred to Stata 17 (College Station, TX) to allow for a structured exploration of codes within the context of educators’ beliefs regarding race. Codes were grouped into themes. A preliminary list of codes was developed by JF and EO following a review of survey responses, and codes were refined through the analytic process. The final list of codes was agreed upon by all three reviewers (JF, CB, EO). Triangulation was achieved through independent coding by the study authors [9]. Two reviewers (JF, CB) coded the first 10 responses together to assure a similar approach to coding. The remaining responses were coded independently by JF and CB with discrepancies resolved by consensus or a third reviewer (EO). After coding was finished, EO read all free text responses and codes and solicited further discussion between JF and CB regarding responses coded in a manner to which EO disagreed. Monthly meetings were held to discuss and refine codes and to identify themes that encompassed groups of codes.

## Results

Of the 147 respondents, 130 (88%) explained why race was important in clinical care. The majority of the 130 participants identified as White (68.5%), Not-Hispanic/Latino/Spanish (94.6%), female (52.3%), and clinical faculty (75.4%) (Table 1). Six categories were identified amongst the responses and were grouped into two themes (Table 2). Categories were contextualized by the extent to which educators believed race was biological (Tables 3 and 4). The coding agreement between the two primary coders was 92%. There were 11 responses for which an additional review was requested by EO, resulting in a change in 7 codes.

**Table 1:** Demographic characteristics of study participants, Georgetown University 2020

Characteristic	N	%
**Faculty**	130	100
Clinical	98	75.4
Preclinical	14	10.8
Both	18	13.8
**Gender**		
Female	68	52.3
Male	60	46.2
Other	0	0
Omitted	2	1.5
**Race**		
White	89	68.5
African American or Black	13	10
Asian	14	10.8
American Indian or Alaska Native	1	0.8
Multiracial	7	5.4
Other	6	4.6
**Ethnicity**		
Hispanic/Latino/Spanish	7	5.4
Non-Hispanic/Latino/Spanish	123	94.6

**Table 2:** Categories and representative quotes from free-text responses of study participants, Georgetown University 2020

Theme	Category	Free-text examples
	Disease risk, Diagnosis & Screening	“ASCVD risk calculators use race to calculate 10-yr risk…HbA1C results in an African American patient need to be interpreted with caution if utilizing for screening for DM…GFR and Cr normal value ranges are different for differ races. Same for screening PSA values, and WBC count”
1		“Certain pre-/disease conditions, such as sickle cell or BRCA-related breast/ovarian cancers, occur predominantly in people of particular ethnic/racial backgrounds. To ignore or minimize these realities would be a disservice to affected individuals of those backgrounds”
	Treatment of Disease	“Individuals of a certain race may respond better to a specific class of medications (Ex. CCIs are more effective for African Americans when treating HTN)”
1		“Need to understand if people of some races have a propensity to toxicities from certain treatments
	Patient Counseling	“If a disease, such as HTN or DM is more prevalent in a population, whether by race, or geographic distribution. More attention should be paid toward educating the patient”
1		“Genetic predisposition to scar has a racial component - understanding that is important in counseling patients about expected outcomes”
	Acknowledging Racism	“It is important to consider self-identified race when considering the impact of structural and systemic racism as an environmental stressor. Also, given the historical trauma that certain communities have been through (e.g. Tuskegee experiments, covert sterilization of Black men and women, etc.), it is important to understand how this may have shaped the relationship between those communities and the medical establishment.”
2		“It is important because it has been well documented now that race has a large role in clinical outcomes. Patients with the same disease but different races will experience vastly different interactions with medical professionals. As such, race has to be involved in making clinical decisions in order to start the process of reducing the influence of unconscious and conscious bias”
	Understanding Socioeconomic factors	“Helps to understand other barriers to care/ability to complete care plans”
2		“Not all groups have same access to health care, insurance, drugs/therapies”
2	Acknowledging Cultural Factors	“Understanding how cultural identification influences understanding & compliance with therapy”
		“Pain is described very differently in certain cultures”

**Table 3:** Responses categorized by themes and perceptions of race as biological

To what extent is race biological?	Clinical importance of race	
	Theme 1: Disease-oriented *N* = 95	Theme 2: Contextualization *N* = 48
Not at all	9	11
Very little	16	9
Somewhat	16	12
Moderate	16	7
A great extent	38	9

**Table 4:** Responses categorized by codes and perceptions of race as biological

To what extent is race biological?	Disease risk	Treatment	Counseling	Acknowledging racism	Socioeconomic factors	Cultural factors
Not at all	5	4	1	8	2	1
Very little	11	10	0	7	3	2
Somewhat	10	7	1	1	8	5
Moderate	11	6	2	4	0	3
A great extent	26	18	1	5	5	3
Total responses	63	45	5	25	18	14

Theme 1: Educators believed race was race is important for understanding and managing diseases.

### Understanding disease risk

The most common theme (*n* = 63) was that race was useful in understanding patients’ risk of disease. Several faculty members described racial differences in disease prevalence, which some attributed to racial differences in genetics. For example, one faculty member stated, “Certain medical conditions are more likely to occur in different racial and or ethnic groups, such as sickle cell or fibroids in African Americans [sic.], BRCA gene in Ashkenazi Jews, endometriosis in Caucasians.” Several faculty members described tailoring disease screening and differential diagnoses by race. For example, participants reported the need to conduct early prostate cancer screening for African Americans and use tighter BMI ranges for Asian patients.

Diseases most described as having a racialized risk were sickle cell (*n* = 13) and hypertension (*n* = 9). For instance, an educator wrote “Black patients are more likely to have essential hypertension at a young age, so that the threshold to look for secondary causes of HTN is lower for non-black patients than for black men, for example.” The need for racialized screening (*n* = 8) was most frequently associated with prostate and colon cancer (*n* = 5). One respondent stated “It is important to know when cancer screening procedure recommendations or medication decisions may vary by race. For instance, African American people should begin colon cancer screening at 45 years old while for White and Asian people screening is recommended to start at 50 years old.” Other diseases described as having racialized risk included breast cancer, ovarian cancer, sarcoidosis, thalassemia, scleroderma, secondary hypertension, endometriosis, cystic fibrosis, Rh isoimmunization, hemolytic diseases, G6PD deficiency, hereditary hemochromatosis, thrombophilia, renal disease, Tay Sachs, Gaucher’s disease, angiotensin-converting enzyme inhibitor associated angioedema, hepatitis B, gestational diabetes, and cardiovascular disease.

For one educator, race and generational status were important to consider. In describing how race could be helpful in care, this faculty member wrote “Asian Americans might be more likely to have HBV infection because their parents might have immigrated from Asian countries where HBV is endemic and might have vertical transmission.” As such, for this individual, race was associated with immigration status and, therefore, disease risk.

A greater share of participants who viewed race as biological, compared to those who reported that race was not biological, provided examples of racialized disease risk. Fifty-nine percent of respondents who described race as extremely biological, compared to 26% who described race as not biological, mentioned the importance of using race to consider disease susceptibility. Among the five participants who found race important to consider for disease risk yet also reported that race was not biological, two provided responses that suggested biological differences between racial groups. One wrote that race was important in rare instances such as considering the risk of sickle cell disease while the other stated “When thinking of inherited diseases. A patient’s heritage might play a role” as example of why race was necessary to acknowledge when considering disease risk.

### Selecting medications and guiding treatment approach

Race was often cited as useful to determine appropriate pharmacotherapy (*n* = 45). Medical educators detailed how race impacts the selection, anticipated response, and risks associated with medical treatment. For example, statements such as “medications have different pharmacokinetic *[sic]* properties depending on race” and “need to understand if people of some races have a propensity to toxicities from certain treatments” were given as explanations for why race was important in care. Additionally, racial differences in treatment intensity were also described with one educator writing: “In scleroderma, African American’s (blacks) have a worse prognosis and have higher risk of dying from lung disease. It is imperative that we are more aggressive with treatment in these patients.” Some responses noted a lack of robust research examining treatment outcomes amongst different racial groups, casting doubt on the applicability of current research findings to racialized patient populations. As one faculty member stated, “most health studies provide data on ‘White’ populations but do not explain variations in clinical outcomes for those of a different race.”

Selection of antihypertensive medications was the most common explanation for why race was important in treatment (*n* = 17). Educators stated, “for hypertension certain patient populations respond better to thiazide diuretics and calcium channel blockers versus ACEi or ARB’s.” Other explanations to adjust medication management based on race included: epilepsy (“for a person of Asian heritage”), heart failure (“black patients have been shown to have lower mortality when taking a medication called Bidil”), multiple myeloma (“venetoclax that is a bcl-2 inhibitor that may be more effective in AA”), and lupus nephritis (persons “who self-identify *[sic]* as black have a better response to rituximab”).

Four faculty who did not believe race was biological, provided explanations that supported the use of race for pharmacotherapy. These faculty members were distinct from the five discussed in the previous section (i.e., who believed race was relevant in considering disease risk). All four provided statements that implied that race may have biological implications, by relating race to treatment response. One such participant penned: “Race may *[sic]* be important in treatment of hypertension in blacks due to studies that have shown more improvement in blood pressure with the use of thiazide diuretics and calcium channel blockers.” Another wrote, “If our evidence-based *[sic]* research is based on certain populations that exclude certain groups, can we really be confident that our treatment recommendations are appropriate for those excluded groups?”

### Counseling and educating patients

The least commonly noted reason for using race (*n* = 5) was for counseling patients. Faculty wrote broadly about the benefits of using race to provide individualized patient counseling and education. Faculty mentioned using race to identify high-risk patients who would benefit from education aimed at mitigating their disease risk and counseling regarding the genetic causes of disease to which these patients may be susceptible. One faculty member discussed the racial disparity of stroke rates stating, “I am a stroke doctor, so for example, African Americans have twice the rate of stroke and twice the mortality. Therefore, even more time should be spent on patient education to change modifiable risk factors.”

Theme 2: Using a patient’s race helped educators contextualize patients’ life experiences and perspectives regarding health.

### Acknowledging patients’ experiences with racism

Many faculty (*n* = 25) believed that accounting for their patients’ race allowed them to acknowledge, and mitigate, the impact of racism on their patients’ lives. Faculty noted structural, institutional, and interpersonal racism as factors that affect their patients’ health. Patient mistrust, attributed to historical mistreatment, exploitation, and abuse, was also mentioned. As one faculty reported, “It is likely more important to recognize racial disparities and implicit biases amongst healthcare professionals. For instance, if an African American (AA) patient is 70% less likely to be recommended for kidney transplant than a Caucasian patient with the same clinical numbers, the provider must recognize this bias and actively work against it when working with an AA patient.” Another provider stated, “Considering race in clinical decision making helps with understanding how racism (for instance, against a person who is black or brown): informs who enters care, creates barriers to optimal health, impacts a patient’s trust in the system or me as a provider. Being cognizant of race also helps me as a provider internally identify my own biases so that I can work to mitigate them.”

Educators who did not believe race had any biological significance had the greatest representation in this category. Of the 19 participants who reported that race was not biological, eight (42%) provided responses in this category. Of the 25 comments categorized as acknowledging racism, 31% were represented by people who believed race was not biological.

### Understanding socioeconomic risk factors

Multiple faculty (*n* = 18) associated race with socioeconomic status. In their written responses, faculty associated race with health literacy, healthcare access, and exposure to environmental hazards. For example, one faculty member answered, “race is a factor in health including exposure to toxins, ability to finance proper diet and healthcare, and delivery of healthcare.” Another stated “Race is just another word for socioeconomic status. It may be easier to use ‘race’ to know the health disparities exist between ‘black’ and ‘white’ than ‘poor’ and ‘rich’ or with health insurance and without.” Many faculty (*n* = 9) emphasized the importance of understanding the barriers that prevent patients from accessing healthcare before making assumptions about treatment adherence and a patient’s interest in improving their health.

### Acknowledging the influence of cultural factors on health

Several responses by faculty (14 responses) acknowledged variations in cultural beliefs and practices amongst different racial groups, impacting how patients conceptualize personal health and view medical care. One faculty member stated, “It is important to take into account an individual’s lifestyle and cultural beliefs when discussing management options as it can be variable based on race.” Furthermore, many comments recognized cultural issues as barriers to care, such as differences in language, a lack of trust in healthcare providers, a preference of homeopathic remedies over pharmacological treatment management, and a stigma against illness.

## Discussion

Medical educators possessed varying views on the clinical utility of race. Many believed race was important for understanding disease risk and **treatment,** often using language that suggested biological differences between racial groups. Medical educators also used race to contextualize their patients’ lives, believing that it provided insight into their patients’ socioeconomic status, cultural practices, exposure to racism, and relationship with the healthcare system. Among educators who did not see race as biological, most explanations given fell under the theme of understanding their patients’ experiences, attitudes, and behaviors. Even among this group of faculty, many gave responses that suggested that race had biological significance.

The themes elicited from our study aligns with findings from prior qualitative work [6, 10]. However, unlike prior studies, our participants reported greater awareness of their own biases. In the Hunt et al. study of primary care clinicians [6], some participants (Black clinicians and a White parent of Black biracial children) discussed the relationship between racism and health outcomes, but none noted how their own racism contributed to racial inequities in health. In our study, several medical educators discussed structural racism—defined as past and present laws, policies and practices that contribute to racial inequity in all aspects of society [11, 12]—and expressed the need to understand how their racial biases affect patients, believing that it was important to remain aware of their patients’ race to counteract their own implicit (i.e., unconscious) biases. This belief is supported by studies on color-blind racial ideology—considered an ultramodern form of racism in which one actively ignores differences between racial groups to impede discussions and remedies of racial inequity—which have found an association between racial prejudice and this ideology [13]. It should be noted that our survey was sent out during the summer of 2020 amid the Black Lives Matter (BLM) movement [11]. Study participants may have responded differently if this survey was administered at a different point in time. The presence of faculty writing about structural racism and implicit bias may reflect the social climate and a temporarily increased acknowledgement of racial inequity.

Nevertheless, despite greater recognition of the structural factors that contributed to racial disparities in health, we still found that many medical educators believed that race had biological relevance and cultural significance. Statements describing the relevance of race to disease screening and treatment were linked to explanations rooted in biological differences between racial groups. This finding agrees with prior work demonstrating that most physicians perceive race as being at least partly genetic [14, 15], and aligns with prior analysis demonstrating that lecture materials frequently suggest biological differences between racial groups [7]. However, our work also stands in contrast to Ibrahim et al.’s finding that educators described racial groups as primarily social groups. This may be because the Ibrahim study limited their analysis to preclinical faculty, a small subset of our study. It is also possible that educators may have provided a description of race that conveys it as a social variable while also holding the belief that it is biologically relevant, as racial differences in disease incidence do exist.

As noted in prior research [6, 10, 16], educators associated race with culture, and, therefore, beliefs and values regarding health. Notably these cultural differences were not described as resulting from structural factors that shape behavior and preferences. For example, racial differences in dietary practices can also reflect differences in food access [17], and not just a preference for specific food types. We also found responses associating race with health literacy. Due to the nature of these comments, we were not able to hypothesize if respondents believed the relationship between race and literacy was due to structural factors that create inequities in education access [18] or assumptions about cultural values regarding literacy and education. Nevertheless, associating patients’ race with assumptions about their cultural health beliefs and values could be due to stereotyping and requires further thoughtful analysis.

### Strengths and limitations

There were several strengths to this study. To our knowledge, this is the first study to investigate medical educators’ perception of the clinical relevance of race, a group whose opinions are particularly relevant to curricular reform efforts, and contextualize these perceptions within their beliefs of race as a biological phenomenon. This study also adds to the few, and mostly dated, studies examining how clinicians value race in clinical care. Finally, this study was relatively large qualitative study, allowing us to find a range of responses from study participants, including new perceptions on physicians’ implicit racial bias that have not been explored in prior literature.

There were also several limitations. First, our study was conducted at a single center and thus findings may not be representative of faculty at other medical institutions. Second, this study was also conducted at the height of the BLM movement which may have resulted in social desirability bias, skewing participants responses. Third, participants were not asked to describe all reasons they believed race to be important. Therefore, we may not have captured all circumstances in which educators find race relevant. Finally, similar to prior published survey studies on race [15, 19], this study had low response rates, likely reflective of the difficulty in getting clinicians to candidly discuss their perspectives on race. However, this was a qualitative study whose purpose was to explore the breadth of reasons medical faculty use race in care, reducing the significance of the low response rate.

## Conclusions

Overall, medical educators found race relevant in disease management (disease screening, prevention, and treatment) and in gaining a comprehensive understanding of their patients’ social environment, including accounting for the harms of structural racism. Their perceptions of the clinical utility of race revealed the belief that race had biological, cultural, and social importance.

Efforts within medical education to teach race as a sociopolitical construct will require directly addressing beliefs regarding its biological and cultural significance. Understanding human diversity from a global, instead of national, perspective can shine light on the inherent contradiction of United States (U.S.) racial categories as it relates to disease risk. Our study indicates that diseases that would benefit the most from an international perspective are sickle cell and hypertension. While sickle cell disease (SSD) is presumed to be a Black disease in the U.S., the prevalence of SSD varies across sub-Saharan Africa. South Africa, Botswana, Ethiopia, Eritrea and Somalia have lower incidence of SSD than the U.S., Spain, Portugal and United Kingdom [20]. Notably, Ethiopians are the second largest group of sub-Saharan Africans in the U.S [21]. While Black Americans, who have significant ancestry from malaria-endemic Western Africa—where sickle cell trait acts as a protective factor against severe malaria infection [22]—have a higher prevalence of SSD than White Americans, that does not make SSD a Black disease [23]. Likewise, examining hypertension from an international perspective reveals that Eastern and Central Europe contain populations with some of the highest rates of hypertension in the world [24]. Considering race from an international lens may also disabuse educators of the notion that people within a race share a culture, thereby reducing essentialist associations between the “cultural values” assumed of different racial groups and health outcomes.

Nevertheless, race, as a representation of inequitable allocation of advantages and disadvantages, is important to health education when contextualized [25]. It is encouraging that some medical educators recognize the harms of structural racism and are aware of the role of provider bias in contributing to poor patient outcomes. Education that elucidates the relationship between structural determinants and poor health can help educators reconcile the belief that race is both socially constructed and clinically relevant [26]. Efforts to remove race as a biological construct should be coupled with efforts to include discussions regarding the impact of structural racism on health [27, 28]. Providing education on, and supporting the development of, tools and measures that explicitly connect structural racism to negative health outcomes, such as the Structural Vulnerability Assessment Tool, and training educators on how to integrate these tools into their instruction will benefit both educators and learners [29].

Moreover, although racial inequities in health are omnipresent, there are geographic variabilities in the magnitude of these inequities [30]. For example, scholars have found associations between living in historically redlined neighborhoods (an explicitly racist federal housing policy that rated the quality of neighborhoods as inversely proportional to the percentage of Black people living there) and cardiovascular health [31, 32]. Learning about the geographic variability in structural racism and discussing how policies and practices contribute to local levels of structural racism and racial health inequity may help educators better understand, and therefore teach, racialized disease risk as resulting from societal factors and not from innate biological or cultural difference.

Education on structural racism should incorporate lessons on the historical construction of racial groups [4, 33]. Racial groups are inherently hierarchal and stem from social and political dynamics. For example, in Virginia, between 1866 and 1924, Black people (formerly described as “colored”) were defined as individuals with 1/4, 1/16, then any African ancestry [34]. Consequently, a person—without Native American ancestry—could transition from White to Black over time. These laws were not enacted in a distant past. In Louisiana, until the 1980s, Black people were legally defined as those with at least 1/32nd African ancestry [35]. Understanding this reality may help educators realize that racial groups are not natural in origin.

In addition to reimagining how educators interpret racial differences in disease risk, more needs to be done to address approaches to knowledge acquisition and retention. Medical knowledge is extensive and expands exponentially, and clinicians rely on mental shortcuts, called heuristics, to help in the decision-making process. However, identity-based heuristics (such as considering cystic fibrosis only among White individuals) likely leads to misdiagnosis, categorical thinking of population groups, and fuels the belief that racial groups are biological groups [36–38]. While it is important to be able to quickly diagnosis patients and understand differences in disease risk, it is better, and more accurate to rely on symptomatology, family history, and contextual knowledge instead of social-determined identity. Heuristics defined by symptomatology or that incorporate the societal factors (e.g., structural racism) should be further encouraged and developed.

Finally, medical educators are also consumers of medical literature, which is infused with analyses, algorithms, and recommendations that incorporate race. Educators must be able to critically engage with this information and teach medical students how to do so too. It would be beneficial to provide educators with guidance on how to approach current race-based literature using tools such as Critical Appraisal of Race in Medical Literature [39] or Critically Analyzing Race in Research [40]. Greater efforts should be made to make tools such as these publicly available, accessible, and easily implementable across multiple medical institutions.

## Data Availability

The data used for the current study are available from the corresponding author by request.

## Abbreviations

AA: African American
DM: Diabetes Mellitus
HTN: Hypertension
BLM: Black Lives Matter
US: United States
SSD: Sickle cell disease
